# The categorisation of hearing loss through telephony in inter-war Britain

**DOI:** 10.1080/07341512.2019.1652435

**Published:** 2019-09-03

**Authors:** Coreen Anne McGuire

**Affiliations:** Department of Philosophy, University of Bristol, Bristol, UK

**Keywords:** Telephony, disability, hearing loss, users, standardization, British Post Office

## Abstract

The telephone in inter-war Britain was an important tool in both the identification and categorisation of individual hearing loss. Between 1912 and 1981, the British Post Office had control over a nationalised telephone system. Linkage between telephony and hearing has long been noted by historians of sound and science, and Post Office engineers in the inter-war period had considerable expertise in both telecommunications and hearing assistive devices. This article first demonstrates how the inter-war Post Office categorised different kinds of hearing loss through standardizing the capacity of its users to engage effectively with the telephone, and secondly investigates how successful it was in doing so. By utilising the substantial but little used material held by BT Archives, we can trace the development of the Post Office’s ‘telephone for deaf subscribers’ and explore how it was used to manage and standardise the variability of hearing and hearing loss within the telephone system.

## Introduction

The ability to hear normally was both defined and moderated by the telephone during the inter-war years in Britain. The telephone was patented by the Scottish/American inventor and teacher of the deaf Alexander Graham Bell in 1876, and it soon became a tool for people with what the Post Office constructed as normal hearing to communicate with each other. It was thus a purely aural device that served to further isolate people with limited hearing from key areas of everyday life. The telephone was domesticated after the First World War had accustomed a generation of soldiers to its use, and it became an essential business tool during the inter-war years. During this period, the telephone was transformed for many users from a luxury item to a necessity, and the ability to use the telephone became a social requisite. For society in general and particularly within the Post Office telecommunications department, the ability to use the telephone (whether amplified or not) meant inclusion in the hearing world. The telecommunications department of the Post Office exemplifies an office hidden behind its role as a cog driving the larger Post Office ‘Government Machine’, with its role in providing a telephone for people with hearing loss ‘marked by opaqueness and discretion’.^1^ It was this department that mediated complaints about the audibility of the telephone, and liaised with the engineering department at the Dollis Hill Research Station to guide possible improvements to the telephone service. This article will demonstrate that the ability to use the telephone was contingent upon, and indicative of, ‘normal hearing’, even though changing standards and improvements to the telephone system meant that the threshold for such categorisation was unstable. These developments are not considered in isolation but rather very much as a product of their time, and especially of the social and cultural milieu of the inter-war years that expedited the nascent welfare state. Rather than seeing the inter-war years as a period of escalation towards the Second World War, after which real change to welfare in the UK began, I show here that major changes were precipitated in the aftermath of the First World War.

I begin the first section of this paper with an explanation of the significance of the Post Office’s ‘Artificial Ear’ technology, which was used by the developing field of audiometry to construct normalcy limits and define the zero line (the normal threshold) on audiograms for testing hearing.^2^ A mechanical ear was initially designed by the National Telephone Company in 1908 to be an audibility testing device, and this design was modified in 1928 by the Post Office to represent a standard ear, which would allow the Office to test their system for the minimum standard of efficiency necessary for telephonic communication. If one’s hearing capacity did not meet this standard, the Post Office offered amplified telephones as part of a service they termed a ‘Telephone for Deaf Subscribers’. This telephone technology is the central focus of this article and is fully analysed in Section Two.

The history of the amplified telephone in the UK has hitherto been almost entirely unexplored. With the exception of some specialised audiological journals, this technology has not been given any attention by the medical or historical professions.^3^ Indeed, historical accounts of general telephony and its provision by the Post Office are sparse.^4^ Perry’s ‘delay’ thesis, which posits that the Post Office and the Treasury worked to delay the provision of telephony in Britain, has recently been challenged by Kay.^5^ With the exception of Kay, these accounts give little consideration to the way the telephone was taken up by the public. As Pool puts it: ‘social scientists have neglected the telephone not only along with but also relative to, other technologies’.^6^ Although Pool’s 1977 account attempts to address this neglect of telephony, it provides only a brief interpretation of the British experience, focusing mainly on the US. Moreover, it uncritically accepts that telephony in Britain lagged behind telephony in the US.^7^ In contrast, the final section of this article (Section Three, ‘Transatlantic Conversations’) uses Mills’ analysis of the connection between deafness and telephony in the US to demonstrate that comparison between the history of the telephone in the US and the UK can illuminate how the nationalised context of British telephony impacted its development and its consideration of ‘deaf subscribers’.

These so-called deaf subscribers did not wait for the Post Office’s consideration, but rather were actively involved in developing amplified telephone technology.^8^ Indeed, the broad connection between deafness and sound technologies has been noted by multiple historians of sound. Since Schafer’s conception of the soundscape in 1977, such historians have increasingly focused on how aurality affects our lives.^9^ Jonathan Sterne’s *The Audible Past* is a classic of this kind, and it explores the connections between attempts to make deafness visible and the creation of sound technologies. Sterne explains that deafness was integral to the development of auditory technologies, from telephony to phonography.^10^ Indeed, people with hearing loss have long been disproportionately involved in the creation of auditory prostheses.^11^ As we will see in Section Three, the concept of deafening was integrated into the telephone system through the activism of people with hearing loss.^12^ Similarly, in the UK, ‘deafened’ individuals were not passive to medicalisation, rather, telephone subscribers with hearing loss adopted various active strategies to ensure they had access to telephony.

**Figure 1.**
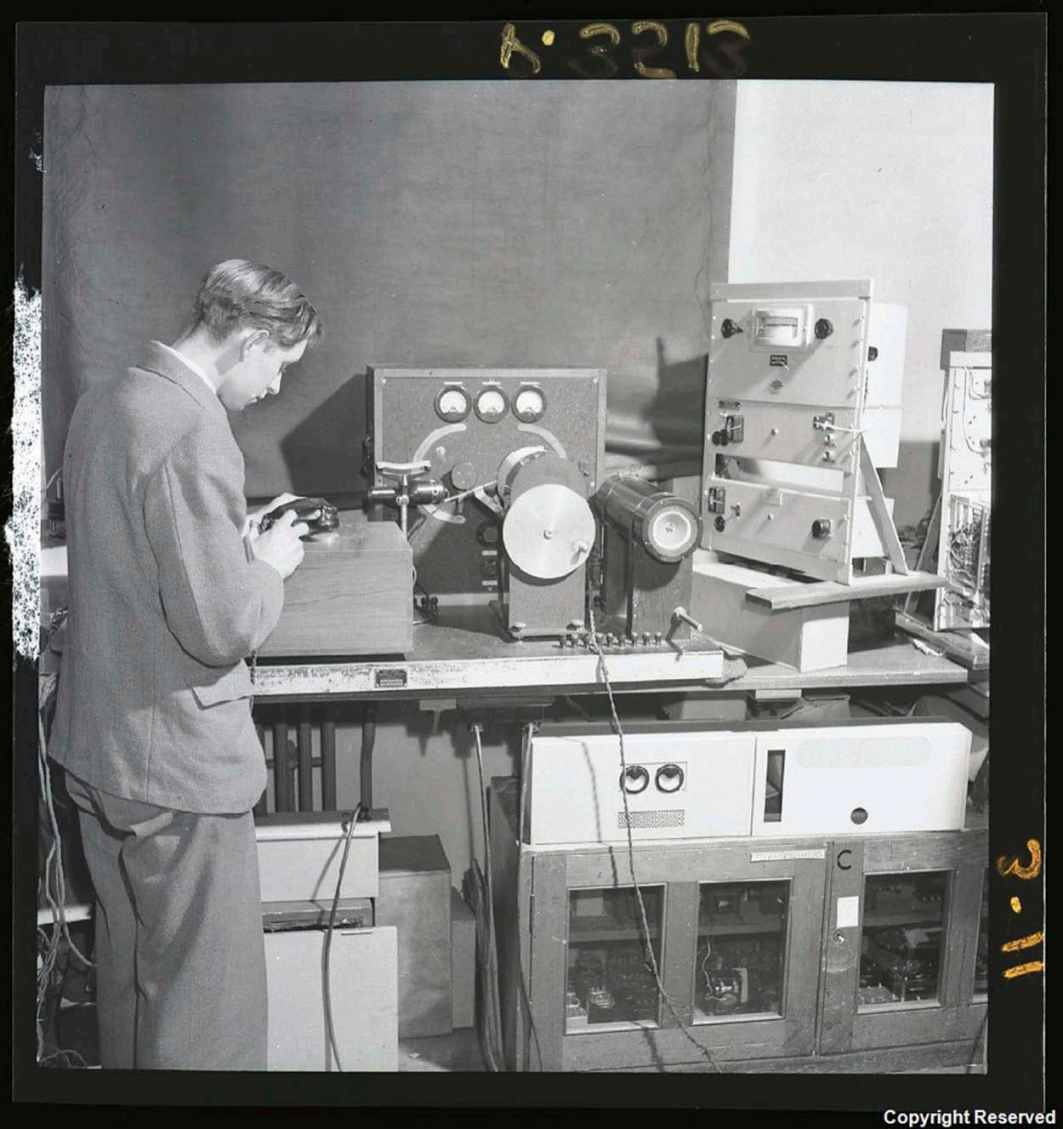
‘Artificial Ear – PO Research Station, Dollis Hill,’ Records created and used by the Post Office telegraph and telephone service 1854–1969, TCB 473/P 3513, British Telecomm Archives, London, England.

This analysis is relevant to historians working in disability studies who have critiqued medicalised accounts of disability because it demonstrates the role of technology in producing disability. Furthermore, it highlights the agency of the disabled user and prioritises their role in influencing technology. Such an investigation is also relevant to those in medical history who have questioned the loss of the patient’s voice in traditional medical history because this reading allows for a broader interpretation of patients as users.^13^ It is however, problematic to refer to amplified telephones as ‘medicines’ or to their users as ‘patients’ because the technology’s status as such was often in flux, and it may be more useful to regard the amplified telephone as being now at a stage of ‘interpretive flexibility’.^14^ The amplified telephone possessed a hybrid status as a unique prosthesis, neither purely medical nor simply technical. This formulation of the amplified telephone as a prosthetic follows from Ott, Serlin and Mihm’s foundational work on prosthetics, which questions the definition and categorisation of prosthetics in a way I extend here to consideration of the amplified telephone.^15^ The utility of this approach to disability history has been demonstrated by Claire Jones in her edited collection on prostheses in Anglo-American commodity cultures, which takes an inclusive view of prostheses to consider devices external to the body and emphasise the variety of prosthesis function and production.^16^ Such category construction is a key concern of this article, as I show how standardisation of hearing loss, hearing measurement, and testing equipment led to the social exclusion of those who did not measure up to the standardised levels of hearing set by the Post Office and its artificial ear (see Figure 1). Indeed, the Artificial Ear was integral to this process, as this technology worked to categorise and enforce normative standards of hearing. In showing how the technology used in the amplified telephone shaped levels of disability, this history adds consideration of telecommunications technology to the insights developed by proponents of the social model of disability, which links the discrimination and problems faced by disabled people to the society and environment in which they live. The Post Office’s categorisation of ‘deaf subscribers’ links with analysis more recently developed by ‘post-social’ models of disability, by demonstrating the powerful impact that standardised classification systems have had on constructing simplified thresholds of hearing levels. Telephone technology contributed to increased quantification of the human body and the inter-war shift towards mechanised practical measures of hearing.

The idea of using an artificial ear to test the quality of telephone transmissions predates the Post Office, and can be traced back to its predecessor, The National Telephone Company (NTC). This was the Bell and Edison conglomerate that controlled most of telephony in Britain before the 1880 ruling on the 1869 Telegraph Act mandated a nationalised service – instated in 1911.^17^ In 1908 The NTC created a ‘mechanical ear’, which was designed to work in conjunction with an artificial voice. To design the artificial voice, the NTC recorded and measured the frequencies of five women who counted aloud the numbers one to five repeatedly, as was standard for transmission tests. The woman with the most pleasing voice was determined through a canvas of the department, and a professional soprano singer was also enlisted to record what was considered an *ideal* frequency. The final arrangement was measured by an artificial ear that we would think of as more closely resembling a recording device than a replica of a human ear. This allowed for quick and mechanical assessment of the telephone’s transmission quality. Its advantage lay in the fact that the telephone circuit was not interfered with, and it gave comparable results to a ‘human test’ but was 218 minutes faster. The NTC report concluded that: ‘There is thus a saving of 67% in time, in addition to the fact that mechanical testing is of course not nearly so exhausting as speech testing’.^18^

The next available report on this subject was produced in 1928, indicating that the Post Office continued using the National Telephone Company system during the intervening 20 years.^19^ The 1928 report marked a critical change in practice, and in the way that the Artificial Ear was designed and used. Rather than functioning solely as a testing system, the Post Office designed the Artificial Ear to resemble a real ear as closely as possible, replicating the functions of the outer, middle, and inner ear, shown in Figure 1 from left to right. They explained that: ‘the present investigation aims at a quantitative determination of the acoustical impedances of a reasonable number of normal (male) ears over a considerable frequency range’.^20^ This reasonable number consisted of 12 male ears; 10 ‘normal’ ears and 2 ‘abnormal’ ears. The data gathered through tests on the 10 normal ears were used to provide the representative standard of normal hearing. The ‘normal’ ears were tested for the mean and extreme resistances to different frequencies, with the average value used to design the Artificial Ear. However, it is clear that there was relevant information garnered from the ears that did not function as expected, as the two abnormal ears were given further tests to establish their ‘impedance’ values, which meant that the Post Office engineers investigated the extent to which the abnormal ears were able to transmit sound through vibrations:

During the investigation two ears, which were abnormal in that their hearing was known to be below normal, came under observation. In both cases the impedances were found to be abnormal, one giving an exceptionally high value and the other an exceptionally low value of absorption at 1100 cycles per second.^21^

Understanding impedance (the conversion of vibrations) was important in improving the Artificial Ear’s design, as the electrical impedance of the artificial organ had previously been adjusted using a real ear.

An artificial ear was considered by the Post Office to be superior to real ears for testing telephone transmission quality for manifold reasons. Firstly, it gave quantitative data ‘for measurement, in absolute units, of the performance of receivers under their working conditions’.^22^ Secondly, it increased the possibilities of testing volume measurements and comparing different circuits and techniques on that basis.^23^ Most importantly, the Artificial Ear provided a permanent trace, a record that did not depend on consistent reproduction and a large number of tests. Many tests were necessary in any use of real ears for research because of the variability of hearing abilities: ‘wide discrepancies between results with different observers necessitates a larger number of tests and observers in order to obtain a representative average’.^24^ Such a testing process was felt to be particularly problematic because of its subjective nature, therefore ‘the elimination of personal bias by the use of an artificial ear becomes more important’.^25^ Thus, the Artificial Ear was conceptualised as an objective technology that could be used to manage the variability of hearing. The resulting machine designated standards of normal hearing in narrow mechanical parameters, which led to a situation in which those who did not fit with the Post Office telephonic standards were categorised as deaf, and in need of a ‘telephone service for the deaf’. Such telephones were developed during the inter-war years through a series of user-forced innovations. There is not scope in this paper to fully outline the series of confrontations between users who demanded telephones customised for their individual type and level of hearing loss against the aegis of the Post Office’s movement towards standardisation in the inter-war years.^26^ However, it is important to clarify that the Post Office’s creation of the category ‘deaf subscribers’ was related to its duty to provide telephony for all citizens without relinquishing control of their network or equipment. The construction and use of the term ‘Deaf Subscriber’ was itself contrived in order to group people with any hearing limitation together, without considering the wide spectrum of hearing abilities or types of hearing loss. As a result of this, telephone users (such as those with greater hearing loss, different frequency needs or bone conductive hearing losses) were unhappy with their telephone provision and demanded that the institution fulfil its duty to provide telephone access to all types of citizens. In this paper I use the term limited hearing to include the full spectrum of individual hearing and to encompass the experiences of all those who may have engaged with amplified telephony. However, Esmail has identified that, despite initial optimist about its use, telephony was not embraced by the Deaf community.^27^ Therefore, the term ‘hearing loss’ is also used to more accurately reflect the experience of those using amplified telephones during this period as belonging to the new category of those ‘deafened’ from age or noise induced hearing loss, who have experienced this change *as a loss* and tried to recover hearing through technology.

The Post Office had total control over the telephone network. State backing also meant that the Post Office was required to work under the financial constraints of the Treasury and act as an arm of the wider government. Due to its position within the Government, the Post Office developed amplified telephone technology according to its changing relationship with the Treasury, whose priorities regarding welfare shifted simultaneously. However, the state and newly enfranchised public expected the Post Office to provide telephones that could be used by people with some hearing loss. Amplified telephony was developed according to, and alongside, the emerging priorities of the welfare state. The Post Office had obtained legalised control over the telephone service in Britain and it was illegal for private companies or individuals to modify or tamper with its apparatus. Crucially, this meant that private hearing aid companies could not attach equipment to Post Office telephones and people with hearing loss could not fit private telephones for use on their lines. As a result, the Post Office was challenged by aspirational users who desired a telephone that could be used by people with less than perfect hearing. For example, between 1928 and 1934 Mr. Horace Buckley, a schoolmaster and war veteran, continually demanded a cheaper amplified telephone for those who had lost their hearing in the First World War and could not afford high telephone rental on a meagre war pension (which was just under half of that accorded to those who had lost their sight).^28^ Buckley threatened to take legal action against the Post Office because he found the Post Office’s amplified telephone ineffective and unnecessarily expensive. His complaints began in 1928 and were not resolved until 1934, when the Post Office introduced an improved amplifier at a reduced rate. His complaint was considered seriously because he had been deafened in the First World War and emphasised that the Post Office had a duty as a government department to help in such cases. Such public demand led to its initial provision of a ‘telephone for deaf subscribers’.

## A telephone for deaf subscribers

The first telephone designed specifically for people with limited hearing was advertised by the Post Office in 1924 when a brief description of the ‘Repeater Telephonic 9A’ appeared in a press release that described a telephone ‘for the use of “Deaf Subscribers” who experience difficulty in the use of the standard telephone’.^29^ This first amplified telephone (the Repeater 9A) featured a controlling key to turn up the volume or decrease it as necessary, which was stored in a separate wooden box, along with the valve amplifier.^30^ This aspect of its design was later modified, following customer criticism. The desk-based design reflected the imagined needs of the intended business user, but the box was very unpopular with customers, who found it cumbersome and stigmatising.

**Figure 2.**
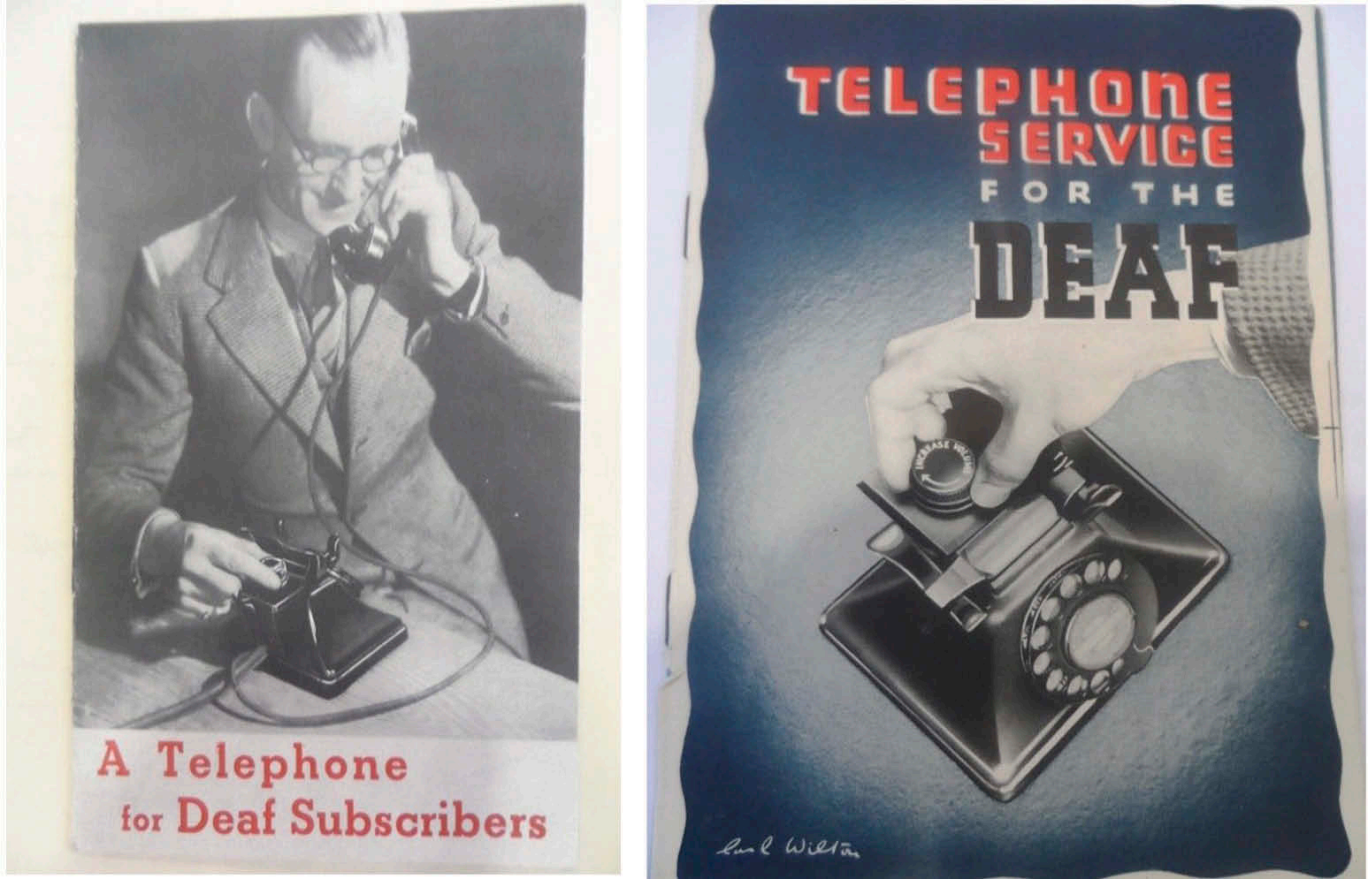
Advertising Booklet ‘A Telephone for Deaf Subscribers’, 1936 (left), and ‘Telephone Service for the Deaf’, 1938 (right), TCB 318/PH 632, British Telecomm Archives, London, England.

Following sustained complaints from customers, an improved amplified telephone (the Repeater 17A) was released in 1934.^31^ This was a cheaper amplifier with a freehand microtelephone. As well as being freehand (meaning the volume control was embedded in the telephone itself rather than in a box), this model used a more powerful valve to boost the signal and increase the volume. However, the integrated receiver (unlike the older candlestick style receiver and transmitter) attracted the ire of users with limited hearing who had been using the older models to listen to the telephone using bone conduction. The Post Office explained that such users ‘had been accustomed to holding the bell receiver to the bone at the back of the ear to obtain best reception for his [sic] particular deafness’.^32^ In response to such complaints, the Post Office created their telephone Repeater 17B, which offered a different frequency characteristic to the Repeater 17A. It was 13.5dbs louder than the 17A and included a tone control button, as can be seen in Figure 2 (right).

The advertisement in Figure 2 (left) was released in a campaign in 1936 to market the amplified telephone as ‘A Telephone for Deaf Subscribers’ and in a revised 1938 copy (right) as: ‘A Telephone Service for the Deaf’. Again, the term ‘Deaf Subscriber’ was itself contrived to group people with a wide spectrum of hearing abilities. During the inter-war period, it was understood that hearing limitations varied in intensity, but understanding of the difference between sensori-neural and conductive deafness was in its infancy. The need for modification of volume at specific frequency levels was not considered by the Post Office until 1936, when a report on ‘Aids to Telephone Reception for Partially Deaf Subscribers’ investigated the possibility of designing an aid which would amplify sound alongside an alternative frequency characteristic.^33^

The Post Office’s understanding of the variability and individuality of hearing limitations was influenced at this point by its collaboration with the medical scientist Dr. Phyllis Kerridge. Her 1935 report on ‘Aids for the Deaf’ in the *British Medical Journal* was extensively cited in their report.^34^ The ‘problem of deafness’ was thus moving from a problem to be solved by engineers into the realm of medicine. However, the principal group targeted by the Post Office in attempts to popularise amplified telephones would not have automatically identified as deaf and may have passed as hearing in all other aspects of their lives.^35^

Those who desired access to telephony in the inter-war years would almost certainly not have recognised the Deaf community and its cultures of the late twentieth century, but less scholarly attention has been paid to those who lost hearing later in life and did not affiliate themselves with the Deaf community. This is in part because there was not an identified community of people with hearing loss, and in part because the stigma surrounding deafness led those with limited hearing to identify as hearing and minimise the significance of their hearing loss. That the stigma attached to hearing loss was high during this time is evident from the rhetoric attached to the advertisements of hearing aids during the inter-war period. Hearing aid companies made exaggerated claims, using vivid language and images to persuade customers of their devices’ effectiveness. The most common trope in such advertisements was to draw on the stigma of deafness to sell their products by emphasising the inconspicuousness and invisibility of the hearing aids.^36^ These advertisements thus relied for their effectiveness on the socially constructed imperative that such disability should be concealed.^37^ This imperative was exacerbated at the start of the twentieth century as stigmatization against deafness increased alongside industrialisation’s demands for standardised practices. As Gooday and Sayer explain, this demand meant that deaf people had to ‘adapt to the hearing world’s oral norms or face marginalisation in unemployment’.^38^

In modern Deaf culture, hearing loss or limitation is not regarded as disabling; rather, the Deaf regard themselves as being defined not by their medical status but through their social and political status.^39^ The point in debating the terminology is to emphasise the spectrum of deaf experience and note that those who would describe themselves as deaf during this period would likely not have used the telephone and those who struggled with it would have described themselves as hearing or possibly as hard of hearing. Crucially, the amplified telephone enabled those using it to ‘pass’ as hearing over the telephone during a period when the stigmatization of hearing loss was high.^40^ The amplified telephone promised to solve issues of both audibility and stigmatization without being apparent to the caller on the other side of the line. Although the amplifying apparatus used in the design of the modified telephone was bulky and visible to its user, it was invisible to the caller on the other end of the line. The invisibility of the amplified telephone as a prosthetic is particularly salient to hearing loss, itself an invisible disability which is often only revealed by the relevant assistive technology.^41^

However, the improved amplified telephone (the Repeater 17B) was £1 more (rental per annum) than the older model. This was not acceptable to users who felt they were being increasingly penalised for their hearing. For example, one subscriber (Mr. Mousley) in 1938 refused ‘to pay any additional rental in respect of it’, and threatened in a letter dated 28^th^ July that if the matter was not given immediate attention he would take the case up with the Postmaster General.^42^ He was especially irate at having to pay £3 at his home residence as well as on his business line and in response he withheld his telephone rent from 9 November 1938.^43^ This was an effective strategy. The Telecommunication Department was concerned and asked the Birmingham Telephone Manager to meet his demands to avoid losing his custom: ‘Messrs. Winn & Co. are good customers, the account being in the neighbourhood of £50 per quarter’.^44^

The Sales Department therefore allowed Mousley a free-of-charge three-month trial of the improved amplifier. However, their real hope was ‘at the end of that time [to] be able to convince the subscriber that the difficulty that he is experiencing is not due to the service but rather to his affliction’.^45^ The Sales Superintendent also pointed out to Mousley that ‘there were a good number of amplifiers existing in the Birmingham telephone area and that he was the only subscriber that complained’.^46^ This statement reveals that the amplification service was popular, although it is unclear whether this was due to widespread deafness or localised problems with the telephones in Birmingham. Contestation of the measurement and categorisation of deafness against the efficacy of the amplifying technology was at the heart of this case. Mousley wrote: ‘I resent very much having to pay for an amplifier at all considering the reason is not really my deafness but the inefficiency of some of the Post Office lines and functions’.^47^ This was disputed by the Post Office, especially when the Traffic Superintendent discovered that Mousley had started to wear hearing aids for ordinary conversation:

Mr Mousley now regularly uses special apparatus with which to carry on his normal business conversation. It consists of a headgear receiver connected to a portable valve amplifier, the power being drawn – I am told – from a 2 volt dry battery. The subscriber carries on a conversation apparently without difficulty when wearing the headgear; but in my opinion he is deafer than ever when not utilising this apparatus.^48^

This dialogue provides an example of what has been described as epistemic injustice of a kind specific to the disabled.^49^ The specialist knowledge that the disabled have regarding how their bodies’ needs are best met has been consistently undervalued, perpetuating a cycle of injustice which undermines the knowledge claims of the disabled.^50^ Not only was the new visibility of Mousley’s hearing loss used to discredit his claims about his inadequate telephone provision, but as well his knowledge about the kind of hearing assistive technology which could have helped him was disregarded.

It is unclear whether this unusual headgear design was provided by a private company or if it was an invention of Mousley himself.^51^ Such innovation was not unusual during this period, and reports of similar designs were outlined in the *BMJ* in 1935. Dr. Kerridge reported on ‘Aids for the Deaf’ and explained: ‘Amateur wireless constructors have often designed very satisfactory circuits for themselves or their relatives by the method of trial and error’.^52^ She described a home-made hearing aid designed by a laboratory assistant who:

… has a quadruple microtelephone instrument, and wears the microphone hidden under his overall. With this help conversation is possible, and he is able to take instructions and keep his job. He uses one battery a week, and finds that the old ones will light his bicycle lamp after they are no good for the hearing aid.^53^

This example gives us insight into the everyday efforts of those trying to use telephone technology to contend with their hearing limitations in this period. It is striking how often such apparatus was characterised by user modification. Another example in this paper was of an amateur wireless constructor who had:

… made himself a valve amplifier set, incorporating a tone control, with which he can hear conversation quite easily […] He finds the tone control satisfactory for clear understanding, and a further advantage is that he can tune out the unpleasant qualities of voices which he disliked in his hearing days.^54^

This selective hearing and use of hearing aids as a means of control has also been noted in the usage of acoustic aids like ear trumpets, which could be powerfully utilised to signal boredom with a conversation.^55^

The development of amplified telephony was marked by tensions between the Post Office’s monopoly and its felt duty to provide a service to citizens with varying hearing needs. The amplified telephone was constructed by the Post Office in a process marked by user input and corresponding design modifications. Improvements to amplified telephony were affected by the complexities of matching individual user needs with the Post Office institutional set up and individuals’ lived experience of hearing conflicted with the Post Office’s desire for standardisation. As a Government department, standardisation was integral to the Post Office’s wider ethos regarding its customers, as providing the same service to all was integral to its democratic position. The aspiration for standardisation was also a built-in component of telephone networks more generally and its pursuit was partially driven by technical necessity. Today, telephony is often used by historians of technology to exemplify how a device can create a network effect because the desirability of the telephone directly correlated to the number of subscribers to the same system.^56^ However, there were tensions between different exchanges and their networks in the era prior to nationalisation in Britain. For example, local subscribers benefited more from local exchanges and public exchanges were more expensive for telephone companies to build than private wire systems.^57^ But different exchanges that offered different types of connection did not fit with the Post Office’s nationalised service ethos. Similarly, though the American Telephone and Telegraph company (henceforth AT&T) did not have a government-mandated monopoly, it still exerted its domination on the lines of communication in a way that has been described as a form of ‘American socialism’, exemplified by the AT&T slogan: ‘One policy, One system, Universal service’.^58^ In opposition to this will towards standardisation, the Post Office’s first amplified telephone did not supply everyone with a telephone that they could use: those with hearing loss too great for this Post Office machine were thus redefined as living on the threshold of ‘deafness’. This meant that users had to actively engage with the technology on an individual level to pressure the Post Office to create an amplified telephone model that fitted with their level of hearing loss (volume amplification) as well as their type of hearing loss (frequency adjustment). Thus, telephone companies created standards of normal hearing outside of the medical sphere. As Mara Mills has explored, this situation was paralleled through the remit of private telephone company AT&T.

## Transatlantic conversations

Mills’ work on AT&T will be used in this final section to provide a transatlantic comparison on the commodification of deafness in the telephone service. Although no single nationalised company in the United States held a state sanctioned monopoly over the telephone service as in Britain, AT&T held a practical monopoly over the telephone system in the US at this time. While AT&T’s monopoly was not legislated by the government, in practice it controlled the telephone service and fought off any competition to maintain its position. One seminal example of its monopolistic powers comes from the 1949–1968 case of the United States versus the Hush-A-Phone company, which centred on the Hush-A-Phone, a device which was attached by the telephone user onto the telephone to improve audibility. This was considered by AT&T to be an illegal attachment that infringed on its monopoly and AT&T went to court to successfully ban the Hush-A-Phone device.^59^ In contrast the Post Office was advised not to press charges in a similar situation involving private hearing aid companies using couplers to link hearing aids with their telephones on the grounds that these companies were not using *physical* attachments. This was an unusual decision because the Post Office operated with a strict blanket ban on any private apparatus on their lines. However, the Post Office did supply amplified telephones for their subscribers with hearing loss throughout the inter-war years, and this was a marked divergence from AT&T’s policy, perhaps indicating a somewhat more inclusive approach towards those with hearing limitations wrought by UK welfare-state ideologies.

AT&T’s specialisation in hearing loss over general telephone lines contrasted with their refusal to provide customers with a telephone system suitable for the deaf, and this became the focus of a widespread campaign in the late 1960s.^60^ However, Bell Telephone Labs did work with the US Public Health Service in 1936 to test the hearing of 9,000 adults using their audiometer.^61^ This allowed for testing of the nation’s audiological health, as well as providing AT&T with more comprehensive data to set the standard of normalcy. Mills explains that AT&T’s study into speech and hearing was wide-ranging and comprehensive, designed for the most efficient telephone service: ‘in the hopes of connecting its system to the average ear, and in turn exploiting that ear’s limitations to establish the requisites for “intelligible” transmission across imperfect lines (and later still, to transmit compressed speech) … ’.^62^ However, Mills points out that because such surveys sought to identify normal hearing and discounted older people and people with hearing limitations, the resulting average was not the norm but rather the upper quartile of the norm.^63^ AT&T sought the average of pre-identified *normal hearing* rather than representing the true variability of hearing ability in the population.

The different contexts of nationalisation versus private development meant that the standards in the UK and the US for normal hearing (the zero line of the audiometer) were different until 1964.^64^ This crucial point demonstrates the subtle influence exerted by the classification systems used in technologies like the telephone on our conception of normal functioning. Comparing AT&T’s services for those with hearing loss to the British Post Office’s service demonstrates how the drive for standardisation was impacted by both local contexts and commercial imperatives. Mills has demonstrated that there were multiple connections between deafness and the development of telephony at AT&T. Firstly, she illuminates that people with hearing loss were activists, and engaged with AT&T in the pursuit of rehabilitation devices.^65^ Secondly, in turn, the novel concept of *deafening* was appropriated by AT&T as both a useful category and an applied term for telephone engineers. Thirdly, AT&T’s audiometric experiments and surveys on levels of normal hearing were utilised in medicine and used to define the ‘normal’ standard of hearing for the audiograms utilised in hearing tests.^66^ That the US norm differed from the UK norm in the inter-war period was demonstrated by the much larger (though still not representative) sample used by AT&T to create the standard. Despite these differences, both the US and the UK telephone companies sought to manage the variability of hearing through mechanisms that promoted a narrow average standard as representative of the norm.

## Conclusion

Designating the standard of normal hearing in a narrow mechanistic fashion using an idealised average resulted in an increased disconnect between the objective measurement of hearing and the subjective correlate. The data used by Post Office engineers in devising the Artificial Ear created the standards that were used to design ‘normal telephones’ in Britain. If users did not have the ability to use the normal telephones, then it followed that they had to use the ‘telephone service for the deaf’. Therefore, these data – fed into the telephone – became the arbiter of a medical condition. Moreover, the National Institute for the Deaf attempted to improve the provision of electric hearing aids sold during the inter-war period by testing them using this machine.^67^ The Artificial Ear was also used in the design of the first NHS hearing aid, the Medresco.^68^ This hearing aid was intended for children and yet data related to children’s hearing was not used in the creation of the Artificial Ear. However, much of this kind of data was gathered by the expanding field of audiometry which used telephones to measure ears literally through audiometers.^69^ There is a clear feedback loop here; between the engineering of the telephone system and the standardisation of hearing integral to audiometric calibration. This loop worked both ways, as deaf ears were used to improve the telephone system and the telephone system was used simultaneously to define and ‘improve’ deaf ears. Moreover, the normative standards embodied in such instrumentation became increasingly invisible as they were perpetuated. Yet, just as Stuart Blume has elucidated: ‘the user “inscribed” in a technology, imagined by its designers, may not correspond with real users in the real world’.^70^

Telephony was ultimately used as a tool in the categorisation of disability by the Post Office. The amplified telephone was used by the Post Office to categorise their users’ identity as hearing (could use the standard telephone model), hard-of-hearing (could use the telephone when amplified), or deaf (could not use the telephone even when amplified). Categorisation largely depended on the efficacy of the technology rather than on the telephone user’s level of hearing. Simultaneously, clinicians used the telephone in the form of the audiometer to create standardised levels of normal hearing and defined deviance from that norm as deafness that could be corrected with appropriate hearing aids.

During the inter-war years, the state of being deaf or hearing became defined through the ability, or otherwise, to use certain kinds of telephone – both literally in the form of the audiometer and socially through the ability to engage with the telephone. To retain their hearing identity and not be categorised as deaf, with the corresponding stigma that invoked, people with hearing loss engaged with amplified telephones. Through such interactions, telephony was used as a tool in the categorisation of disability and, in turn, telephone users modified the technology to fit their personal needs, experiences and identities. Yet this promise of improvement was not realised in practice because the Post Office’s standard amplified telephone model did not reflect either the significant diversity of users’ hearing or the variability of hearing loss. The standardisation of normal hearing and the categorisation of the deafened was therefore both facilitated and created in line with the priorities of the British Post Office’s telephone system. This analysis demonstrates the fluctuating and contingent thresholds of normalcy construction and reveals how deafness was socially and technologically constructed in inter-war Britain.

While a growing number of historians of disability examine the multiple ways in which social contexts shape and define disability and ability, this analysis provides a new perspective on the fluid boundaries between hearing and deafness created by the telephone. This neglected episode of early twentieth-century telephony redefines the relationship between technology, communications, and disability, and broadens our historical understanding of deafness. Science and Technology Studies scholarship has decisively demonstrated that technologies are not neutral, but rather are shaped by the cultures, contexts, and the actors that make them.^71^ By focusing on the forces and norms which enact technologies we reveal the socio-cultural and anthropological decisions embedded within them. This is an issue of key concern to disability studies because of the normativising power of technologies like the artificial ear. As this article has demonstrated, technology’s development is interlinked to the classification and enforcement of normative categories. Such analysis is not only relevant to disability history, but also relates to current concerns about the categorisation of data in healthcare. This is a topic that has recently been given sustained attention by the field of media and communication studies.^72^ However, our veneration of big data relates to a longer history of measurement technology as a form of control. To explore these topics more thoroughly, we need to embrace more interdisciplinary work between media history, medical history, STS, and disability history. Connecting these fields will foster a greater understanding of the social and technological construction of normalcy and the cultural power of technology.

## Notes

1. Agar, *The Government Machine*, 395.

2. Audiograms represent hearing ability measured by audiometers in graphical form. Telephone technology was used in audiograms from the nineteenth century and in 1922 commercial vacuum tube audiometers were used to test hearing. For a history of audiometric testing in Britain see Virdi and McGuire, “Science of Audiometric Standardisation,” 123–46.

3. Castle, “Telephone Communication,” 91–104.

4. See: Crutchley, *GPO*; Robertson, *Story of the Telephone*; Perry, *Victorian Post Office*; and Campbell-Smith, *Masters of Post*.

5. Perry, “The British Experience,” 69; and Kay, “Inventing Telephone Usage,” PhD diss.

6. Pool, “Introduction,” 2.

7. Kay, “Inventing Telephone Usage,” PhD diss.

8. McGuire, “Inventing Amplified Telephony,” 70–90.

9. Schafer, *The Soundscape*. See also: Pinch and Bijsterveld, *Oxford Handbook of Sound Studies.*

10. Sterne, *The Audible Past*, 41.

11. Cf. the original amplified telephones described in McGuire, “Inventing Amplified Telephony,” 80–84, Oliver Heaviside’s work in long distance telephony and radio signals explored in Gooday and Sayer, *Managing Hearing Loss*, 8, and Edison’s experiments with hearing aids in Mills, “When Mobile Communication Technologies were New,” 142.

12. Mills, “Deafening,” 122.

13. Linker, “On the Borderland,” 499–535.

14. Oudshoorn and Pinch, *How Users Matter*. See also: Bijker and Pinch, “Social Construction of Facts,” 17–50.

15. Ott, Serlin, and Mihm, *Artifical Parts, Practical Lives*. See also McLuhan, *Understanding Media.*

16. Jones, “Introduction,” 1–23.

17. The 1869 Telegraph Act granted this monopoly over communications and it was confirmed in 1880 that this Act included telephony even though the telephone had not been invented when the Act was first conceived. See: Campbell-Smith, *Masters of the Post*, 193.

18. Engineer in Chief’s Department, “The selection of suitable substitutes for the human voice and ear in transmission testing,” 26 February 1908, 29. The National Telephone Company, TCB 22 T0339, British Telecomm Archives, London, England [henceforth BTA].

19. The Post Office kept the structure of the NTC and inherited all its outdated equipment because the company stopped investing in its stock when they realised that they would have to hand it over to the government. The Post Office also had to invest enormous amounts of material and research in the war, which would account for this gap in research and development.

20. Alridge and West, “Measurements of the Acoustical Impedance of Human Ears,” Research Report No. 4697 For research at Dollis Hill May-September 1928, 2. TCB 422 04697, BTA. Acoustical impedance was defined in the report as “the ratio of alternating pressure to alternating rate of volume displacement, i.e. if a piston of area is vibrating with velocity v and a pressure p per unit area is developed at its surface due to its contact with the medium (air)”.

21. Ibid., 15.

22. Alridge and West, “An Artificial Ear,” Research Report No. 4946 For Research Station at Dollis Hill, 21 October 1929, 7. TCB 422 04946, BTA.

23. Ibid., 8.

24. Ibid.

25. Ibid.

26. For a fuller discussion of user involvement in the initial development of amplified telephones see McGuire, “Inventing Amplified Telephony,” 70–90.

27. Esmail, *Reading Victorian Deafness.*

28. Hansard, 6 March 1917, vol. 91, cols. 241–354, line 251.

29. Memorandum from Accounts to the Superintending Engineer, 23 October 1924, POST 33/1491C, BTA.

30. Memorandum from the Engineer in Chief (Accounts) to the Superintending Engineer concerning insuring the repeater 9A, 23 October 1924, POST 33/1491C, BTA.

31. McGuire, “Inventing Amplified Telephony,” 74.

32. “Aids to telephone reception for partially deaf subscribers,” Research Report no. 9150, Post Office Research Station, 21 April 1936, TCB 422 09150, BTA.

33. Ibid., precis.

34. See also: Kerridge, “Aids for the Deaf,” 1314–7.

35. Esmail, *Reading Victorian Deafness*. On passing see Cureton, “Hiding Disability and Passing,” 15–32.

36. Stamp book advertisements for hearing aids sent to the Post Office from complaining customers. BPMA POST 33/3481B, British Postal Museum Archives, London, England.

37. Mills, “When Mobile Communication Technologies were New,” 144.

38. Gooday and Sayer, *Managing Hearing Loss*, 84.

39. For an overview of d/Deaf history and the history of hearing loss see Gooday and Sayer, *Managing Hearing Loss*; and Davis, *Enforcing Normalcy.*

40. Brune and Wilson, *Disability and Passing*.

41. For consideration of how this affects user rejection of assistive technology see McGuire and Carel, “Stigma, Technology and Masking,” 1–21.

42. Letter from Mr. Mousley to Telecommunications Department, 28 July 1938, TCB 2/171–2/172, BTA.

43. Letter from Charles Winn & Co to the Service and Sales Manager, 9 November 1938, TCB 2/171–2/172, BTA.

44. Letter from The Telecommunications Department, Telephone Branch, 2 November 1938, TCB 2/171–2/172, BTA.

45. Letter from the Sales Superintendent in the Birmingham Area to the Service and Sales Manager, 30 July 1938, TCB 2/171–2/172, BTA.

46. Memorandum by Acting Sales Superintendent in Birmingham, 17 November 1938, TCB 2/171–2/172, BTA.

47. Letter from Mr. Mousley to the District Manager, 20 April 1938, TCB 2/171–2/172, BTA.

48. Letter from the Traffic Superintendent to the Service and Sales Manager, April 1938, TCB 2/171–2/172, BTA.

49. Scully, “From ‘She Would Say’,” 106–24.

50. Ibid.

51. The letterhead on the Charles Winn company stationary shows that they manufactured valves and indicates that Mr. Arthur Mousley held an MBE. TCB 2/171–2/172, BTA.

52. Kerridge, “Aids for the Deaf,” 1314–7.

53. Ibid.

54. Ibid.

55. Gooday and Sayer, *Managing Hearing Loss.*

56. Agar, *Constant Touch*, 64.

57. Kay, “Inventing Telephone Usage,” 150–80.

58. Sterling, “The Hacker Crackdown,” 37.

59. George, “Federal Communications Commission and the Bell System,” 460–2. Thanks to Mara Mills for highlighting this case to me.

60. This struggle has been documented in Lang, *A Phone of Our Own*. The work also emphasised the difficulty of working with the British Post Office.

61. Mills, “Deafening,” 132.

62. Ibid., 120.

63. Ibid.

64. Noble, *Assessment of Impaired Hearing*, 178–9.

65. Mills, “Deafening,” 121.

66. Ibid., 118–43.

67. The Post Office tested hearing aids at the Dollis Hill Research Station on behalf of the National Physical laboratory and in conjunction with the National Institute for the Deaf. See: Wharry and Crowden, “Correction of Hearing Defects,” 1189.

68. Plans for an NHS hearing aid began in 1947. The Medresco was a contraction of ‘Medical Research Council’, and this name has led to over-emphasis of the MRC’s role in its creation with attendant erasure of the work of the Post Office. Instead of designing a new amplified telephone alongside the NHS hearing aid, the Post Office engineers designed an adaptor to link the new hearing aids with telephone receivers. This would allow users to link into any telephone and not just their home sets. See: McGuire and Carel, “Stigma, Technology and Masking,” 1–21.

69. Virdi and McGuire, “Phyllis M. Tookey Kerridge,” 123–46.

70. Blume, *The Artificial Ear*.

71. For comparative cases see: Winner, “Opening the Black Box,” 362–78, and “Do Artefacts Have Politics?” 121–36. See also: Rosenberg, *Inside the Black Box.*

72. Fiore-Gartland and Neff, “Expectations of Data,” 1466–84.
